# Memory rescue and learning in synaptic impaired neuronal circuits

**DOI:** 10.1016/j.isci.2023.106931

**Published:** 2023-05-29

**Authors:** Kwan Tung Li, Daoyun Ji, Changsong Zhou

**Affiliations:** 1 Department of Physics, Centre for Nonlinear Studies, Beijing–Hong Kong–Singapore Joint Centre for Nonlinear and Complex Systems (Hong Kong), Institute of Computational and Theoretical Studies, Hong Kong Baptist University, Hong Kong, China; 2 Research Center for Augmented Intelligence, Research Institute of Artificial Intelligence, Zhejiang Lab, Hangzhou 311100, China; 3 Department of Neuroscience, Baylor College of Medicine, Houston, TX 77030, USA; 4 Department of Molecular and Cellular Biology, Baylor College of Medicine, Houston, TX 77030, USA

**Keywords:** Cognitive neuroscience, Molecular neuroscience, Neuroscience

## Abstract

Neuronal impairment is a characteristic of Alzheimer’s disease (AD), but its effect on neural activity dynamics underlying memory deficits is unclear. Here, we studied the effects of synaptic impairment on neural activities associated with memory recall, memory rescue, and learning a new memory, in an integrate-and-fire neuronal network. Our results showed that reducing connectivity decreases the neuronal synchronization of memory neurons and impairs memory recall performance. Although, slow-gamma stimulation rescued memory recall and slow-gamma oscillations, the rescue caused a side effect of activating mixed memories. During the learning of a new memory, reducing connectivity caused impairment in storing the new memory, but did not affect previously stored memories. We also explored the effects of other types of impairments including neuronal loss and excitation-inhibition imbalance and the rescue by general increase of excitability. Our results reveal potential computational mechanisms underlying the memory deficits caused by impairment in AD.

## Highlights

• Synaptic impairment alters neural dynamics and hinders memory recall
• Rhythmicity modulates membrane potential to rescue memory from synaptic impairment
• Slow-gamma rhythmicity best modulates potential
• Synaptic impairment induces bursts to impair learning new memory

## Graphical abstract

**Figure:**
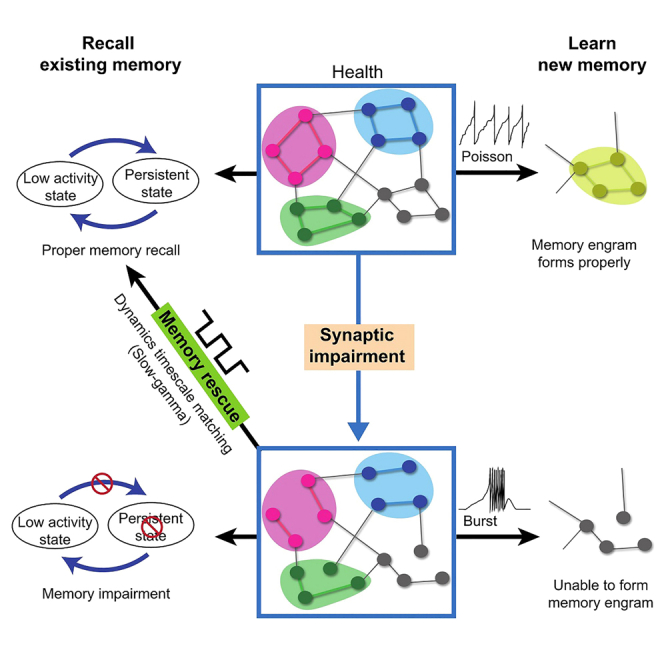

## Introduction

Abnormal amyloid-beta (Aβ) plague deposition is a key feature of Alzheimer’s disease (AD) and they affect brain circuits at different levels.^1^^,^^2^^,^^3^^,^^4^ At the neuronal level, action potential peaks are reduced^5^ and excitability changes.^6^^,^^7^ At the synapse level, synaptic currents are potentiated at low concentrations of Aβ, but depressed at high concentrations.^8^ At the circuit level, Aβ induces synaptic impairment, leading to a reduction in number of neuron,^9^^,^^10^^,^^11^ and the probability of synaptic connectivity between neurons,^2^^,^^12^^,^^13^ which may change the dynamic state of the circuit and affect its responses to external stimuli.^12^ In some mouse models of AD, excitatory neurons are over-excited and the excitation-inhibition (E-I) balance breaks down, leading to deficits in spatial memory performance.^13^ In other animal models, however, excitatory neurons are under-excited and hippocampal slow-gamma (∼40 Hz) oscillations are reduced, which also produces spatial memory deficits.^14^ How these seemingly contradictory results in circuit excitability and their intricate relationship with the impairment of connectivity at different degrees are not clear.

Memory deficits in AD may be a result of impaired memory recall or impaired learning, leading to improper memory storage. Impaired memory recall is likely to be associated with the activation of memory engrams.^15^^,^^16^ Optogenetic activation of engram cells in the dentate gyrus of the hippocampus restores memory recall in an AD model,^15^ pointing to the involvement of improper memory recall in the memory deficits of AD. This is supported by the rescue of memory recall via slow-gamma optogenetic stimulations in another AD model.^14^ Moreover, overlapping of engrams and neurons encoding novel information during recall was found to induce memory impairment in AD.^17^^,^^18^ In addition, it is well known that the formation of new memories during learning is severely impaired in AD and AD-related animal models.^19^^,^^20^^,^^21^^,^^22^^,^^23^^,^^24^ However, it is unclear how these different aspects of memory deficits are related to different degrees of connectivity deficit.

In this study, we aimed to understand how connectivity deficits lead to changes in neuronal activity patterns and the relevant neural oscillations that underlie the impaired recall of existing memories, the rescue of memory recall by slow-gamma stimulation, and impaired learning. Because it is difficult to experimentally monitor specific engram cells associated with different memories and their dynamic changes at the neuronal and synaptic levels, we conducted our study using a computationally simulated neural network model, where the degree of synaptic connectivity could be manipulated and neural firing patterns, their dynamics, and associated synaptic changes could be precisely monitored.

We found that reducing connectivity during memory recall caused a transition of memory-coding neurons from a synchronous state to an asynchronous state, reduced slow-gamma oscillations, and reduced neural activities associated with memory recall. Moreover, the neural activities were first increased and then reduced; suggesting that the seemingly contradictory results in circuit excitability may be induced by different degrees of connectivity impairment. Second, our simulation showed that rescue using external slow-gamma stimulation at low connectivity caused a side effect of co-activating multiple memories, despite an improvement in recall performance. Third, during the learning of a new memory in the presence of existing memories, our results showed that reducing connectivity induced impairment in coding the new memory engram, but did not strongly deteriorate existing memories. Furthermore, our simulation suggested neuronal loss and synaptic weight reduction caused severer damage to memory performance. General increase of excitability might be an alternative measure to partially rescue memory and slightly increasing excitatory to inhibitory current ratio (E/I ratio) at low connectivity might be a remedy to memory performance caused by synaptic impairment.

## Results

### Connectivity reduction impairs recall of stored memories

**Figure 1:**
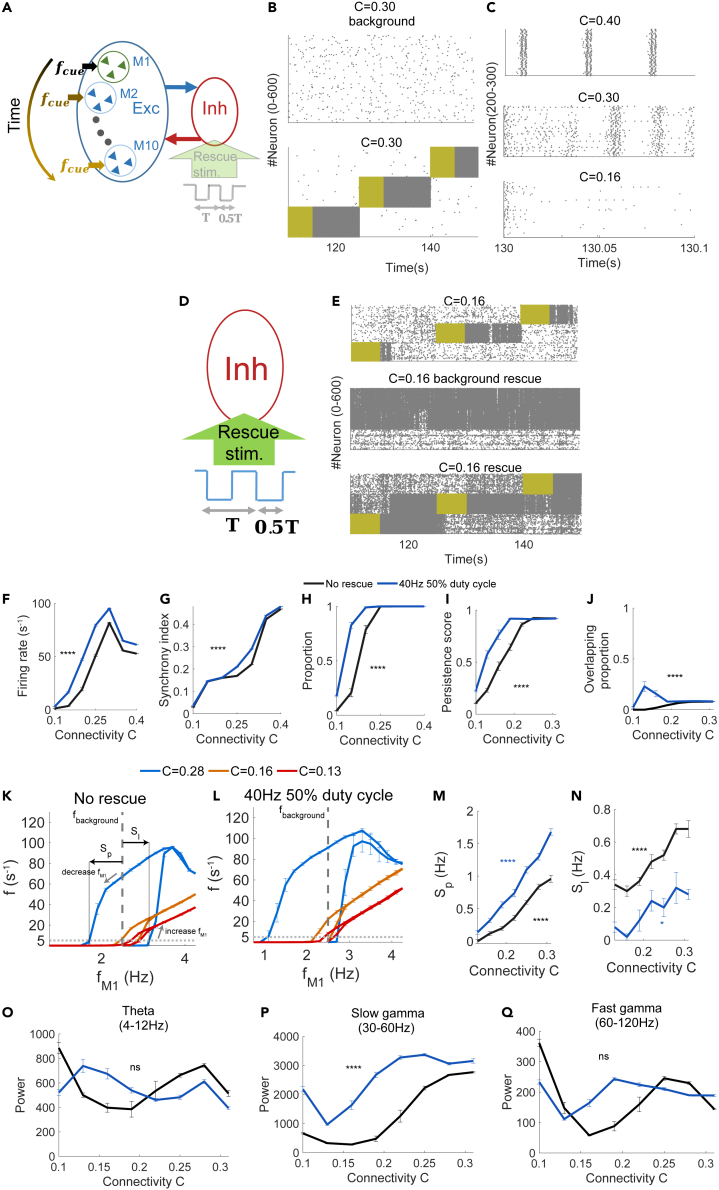
Reduced connectivity impairs memory recall and memory rescue (A) Schematic depiction of the model architecture. (B) Spike raster plots of 400 excitatory neurons (corresponding to two memory engrams) at C = 0.30 in a background state (top) and during memory recall with a strong cue input (bottom). The periods with a strong cue (12.5 Hz) input (5 s) are marked in yellow. Note the intense firing of cued memory neurons (persistent state) even after cue termination. (C) Close-up view of the spike raster plot (100 ms window, 100 neurons in a memory engram) after cue termination at different C values: 0.4 (top), 0.3 (middle), 0.16 (bottom). (D) As in (A), but with memory rescue. Rescue stimulation at 40 Hz (T = 25 ms) with a 50% duty cycle was applied to half of the inhibitory neurons. (E) Spike raster plots of neurons in three memory engrams under different conditions. In this example of memory rescue, C = 0.16 and rescue stimulation (40 Hz, 50% duty cycle) was turned off during the recall with a strong cue input (top), turned on in the background state without the cue (middle), or turned on during recall (bottom). (F–H) Quantifications of cued memory neurons during memory recall with and without rescue stimulation—population mean firing rate (F), synchrony index (G), and proportion of high-firing memory neurons (H). (I and J) Quantifications of persistent states and local field potential oscillations at different C values—persistence score (I), overlapping proportion (J). (K and L) Bifurcation diagram expressed by firing rate of cued memory engram with respect to cue input strength, $f_{M1}$, for various connectivity C, with or without pulses application—No rescue (K), 40Hz 50% duty cycle (L). Firing rate increases followed the right path when cue input frequency increases; decreases followed the left path when cue input frequency decreases. Two intersections point at $f_{M1}=f_{background}$ indicates two steady states coexist at $f_{background}$. Different colors indicate different C. (M and N) $S_{p}$, (M) and $S_{l},($ N) at different C under $f_{background}=2.5\;Hz$. (O–Q) Quantifications of persistent states and local field potential oscillations at different C values—theta power (K), slow-gamma power (L), fast-gamma power (M). In (F-J, M-Q), blue and black indicate with and without rescue stimulation. Data in (F-Q) are represented as mean ±. n = 10 trials/setting. ns: p > 0.05; ∗∗∗∗: p < 0.0001; two-way analysis of variance (2ANOVA) test (F-J, O-Q); one-way analysis of variance (1ANOVA) test (M, N). See also Figures S1 and S2.

We started by studying the effects of reduced connectivity on neural activities related to memory recall. The model we used was a randomly connected conductance-based E-I neuronal circuit^25^ consisting of 2,000 excitatory ($n_{E}$) and 400 inhibitory ($n_{I}$) integrate-and-fire neurons (Figure 1A) to model a local circuit in CA1 in hippocampus. The interneurons in our model were able to receive inhibitory external stimulation to suppress their membrane potential, thus reducing firing for memory rescue by enhancing the excitability of the circuit. This is to model the inhibitory neurons in CA1 receiving manual activation of medial septum parvalbumin (MSPV) neurons as in Etter et al. experiment.^14^ The rescue stimulation was turned off here to study memory recall. We considered a case of 10 stored memories in our model. Every single memory was coded in a preset group of 200 non-overlapping excitatory neurons (memory engram) connected via strong connections, while other connections (between memory engrams) were at a weak baseline level. All connections were randomly assigned a connection probability (connectivity, C). We modeled the synaptic impairment seen in AD by systematically reducing C (see STAR Methods for details).

In addition, we modeled memory recall by applying a strong cue input (12.5 Hz Poisson spike trains), $f_{cue}$, for 5 s to every neuron in a memory engram (relative to a weak 2.5 Hz background input), and the 10 memory engrams were recalled sequentially. As shown in an example raster plot, the background activity in the absence of the cue input was low (Figure 1B top). The cue input often moved (or activated) the neurons in a memory engram into a state characterized by a high level of firing activity that persisted even after the cue was terminated (persistent state), while other neurons remained at a low-activity state (Figure 1B bottom). The persistent state was commonly studied in working memory models of spiking neurons.^26^^,^^27^^,^^28^^,^^29^^,^^30^^,^^31^^,^^32^ Here, we used the cue-triggered persistent states after cue termination as a proxy of neural activity underlying memory recall, because these states are produced by the strong excitatory-to-excitatory connections within a memory engram.^31^^,^^33^^,^^34^ Therefore, to study how connectivity affects memory recall, we examined how neural activities associated with persistent states depended on C.

Based on raster plots at various C values (Figure 1C), we observed a dynamic change in firing rate after the cue input and firing synchrony among neurons in the cued memory engram (cued memory neurons). When C was high (at 0.40, Figure 1C top), cued memory neurons fired at a high rate and their spikes were synchronized and phase-locked to oscillations at a frequency of approximately 40 Hz. When C was decreased to 0.30 (Figure 1C middle), neuron firing rates were increased and became less synchronized and less phase-locked. When C was further decreased to a low value of 0.16 (Figure 1C bottom), the cued memory neuron firings eventually became non-synchronous and decreased to a very low rate.

We quantified this observation by measuring the firing activities of cued memory neurons in a window from the cue termination to 10 s after termination (before the onset of another cue input to another memory engram). The measurements included the population mean firing rate (number of spikes per neuron within cued memory engram per second), the synchrony index, and the proportion of high-firing neurons (those with a rate $>\;5\;s^{-1}$, the threshold to separate a low-activity state from a persistent state; see STAR Methods and below) in the cued memory engram. We found that a mild reduction in C induced a significant increase in the population mean firing rate. However, once the rate reached a peak, it rapidly decreased with reduction in the C value (Figure 1F black line). Therefore, it suggested that the seemly contradictory results in experiments could be caused by different degrees of synaptic impairment in AD. At mild degree of synaptic loss, the neural activity was enhanced, but it was decreased with serve synaptic impairment. In contrast to the non-monotonous change in the population mean firing rate, the firing synchrony (Figure 1G black line) and the proportion of high-firing neurons (Figure 1H black line) decreased monotonously as C decreased, with steep changes occurring at C = 0.25–0.35. Additional analyses found that low C values (< 0.2) led to low firing rates and low firing synchrony in a range of synaptic weights connecting excitatory neurons to inhibitory neurons ($g^{E\to I}$ from 0.10 to 0.20), suggesting that the effects of C were not sensitive to specific parameter values in our model (Figures S1A and S1B). These results indicate that neuronal activities associated with memory recall are compromised as C is reduced; suggesting that reduced connectivity impairs memory recall.

To further characterize recall performance, we next detected persistent states and quantified how their properties depend on C. A persistent state of a cued memory engram was defined as a period after cue input termination when the mean firing rate of its memory neurons within a 1 s window at each 1 ms time step exceeded a threshold of 5 $s^{-1}$ (see STAR Methods for details). This threshold was determined because the firing rates of all excitatory neurons within 10 s after cue input termination displayed a bimodal distribution when C was high (> 0.19), with two groups of neurons separated by a rate of ∼5 $s^{-1}$ (Figure S1C), whereas those with background activity levels did not, with a single group of low-rate neurons (Figure S1D). We quantified each persistent state using two metrics. The first metric was persistence score for each persistent state, which was used to quantify how its duration deviated from the expected length (from the termination of the cue input to one memory engram to the beginning of the cue input to another). The persistence score was between 0 and 1, with a larger value indicating duration closer to the expected length. The second metric, overlapping proportion, was motivated by the observation that engram overlapping can induce memory impairment^17^^,^^18^ and was used to quantify the degree of co-activation of persistent states of more than one memory engram when one of the engrams was cued (see STAR Methods for details). We found that at high C values (≥0.25) the persistence score was close to 1, while at low C values (≤0.25) the score decreased rapidly (Figure 1I). In contrast, the overlapping proportion was always low (Figure 1J). This result suggests that reducing connectivity impairs engram activation.

To obtain insight into the mechanism how the persistence score and overlapping proportion were affected by C, we considered a memory engram M1 and examined the relation between firing rate (f) and cue input applied to one engram M1 ($f_{M1}$) (See STAR Methods for details). As expected for subcritical bifurcation, which is the working principle of persistent activity,^34^^,^^35^ considering M1, starting at low-activity state (far left of the curve in Figure 1K) and increasing $f_{M1}$, the firing rate of M1 followed the right path to enter persistent state; while starting from persistent state (far right of the curve in Figure 1K) and decreasing $f_{M1}$, the firing rate of M1 followed the left path to enter low-activity state, thus the system displays hysteresis loop with two bifurcation points (here measured by firing rate passing $f=5s^{-1}$, gray dashed line) depending on C (Figure 1K). The left and right bifurcation points corresponded to switching from persistent state to low-activity state and from low-activity state to persistent state, respectively. Moreover, for the background input $f_{background}=2.5\;Hz$ considered in our model (vertical dashed line), $f_{background}$ was below both bifurcation points when connectivity is reduced to C < 0.16, thus persistent state did not exist.

As a result, the distance between $f_{background}$ and the two bifurcation points are correlated with strength of signal required to have state switches between persistent state and low-activity state, and we could define the distance between $f_{background}$ and the two bifurcation points, $S_{p}$ and $S_{l}$ (Figure 1K), as stability of persistent and low activity state. Larger $S_{p}$ and $S_{l}$ implies higher stability of persistent state and low activity state, respectively, and thus it requires stronger deviation from $f_{background}$ for state switch to occur. Therefore, larger $S_{p}$ means persistent state can last longer, leading to higher persistence score; larger $S_{l}$ means the low-activity state is not easily switched to persistent state, leading to lower overlapping proportion.

We found that both $S_{p}$ and $S_{l}$ were decreased with C (1ANOVA, $F_{\left(6,63\right)}=103.88,p=9.64\times 10^{-31}\text{,}$ Figure 1M black; 1ANOVA, $F_{\left(6,63\right)}=17.82,p=5.96\times 10^{-12}\text{,}$ Figure 1N black), which implied that persistence score and overlapping proportion were reduced with C. These results provide a computational explanation for the dependence of persistent state on C in Figures 1I and 1J black.

Moreover, given the experimental evidence for the involvement of neural oscillations, especially those with slow-gamma frequencies (30–60 Hz), in memory recall,^14^ we also quantified the power of slow-gamma oscillations associated with memory recall (10 s after cue input termination) in our model, as well as those of theta (4–12 Hz) and high-gamma (60–120 Hz) oscillations for comparison. We found that slow-gamma oscillation power decreased rapidly as C was reduced (Figure 1P), whereas theta and fast-gamma oscillation power were less sensitive (Figures 1O and 1Q). Therefore, our results suggest that the impairment in engram activation at low C values is accompanied by impairment in slow-gamma oscillations.

We focused on connectivity reduction because it is widely reported in research articles about AD.^2^^,^^3^^,^^36^^,^^37^ However, neuronal loss and reduction in connection strength might generate similar effect as the reduction in C. We introduced $R_{N}$, $R_{c}$ and $R_{w}$, where $R_{m}=\frac{m}{m_{0}},m=N,C,w_{j\to i}$ and $N_{0}$, $C_{0}$, $w_{0}$ were constant connectivity, number of neurons and synaptic weight, respectively, fixed to the initial total neuron number $n_{E}+n_{I}$, the connectivity of 0.25, and the initial set of synaptic weight $w_{j\to i}$ in the ciruit model. Thus, $R_{N}$, $R_{c}$, and $R_{w}$ were fraction of remaining neurons, connections, and synaptic strength. We examined how reduced $R_{N}$, $R_{c}$, and $R_{w}$ impaired persistent states and found that they all reduced both persistence score and overlapping proportion, but the effects by reduced $R_{N}$ and $R_{w}$ were stronger than that of reduced $R_{c}$ (Figures S2A and S2B). This is because connectivity reduction may remove plenty of weak inter-engram connections $g^{E\to E}$ with only little effect on strong intra-engram connections $g_{M}^{E\to E}$ ($\frac{\#g^{E\to E}}{\#g^{E\to E}+\#g_{M}^{E\to E}}=0.9$ at C=0.25). In contrast, neuronal loss and connection strength reduction decrease the number of intra-engram connection $g_{M}^{E\to E}$ or the value of $g_{M}^{E\to E}$, respectively.

Furthermore, in an AD mouse model, excitatory neurons are found over-excited and the E-I balance breaks down, leading to deficits in spatial memory performance.^13^ Therefore, we tipped the E-I balance by reducing $g^{E\to I}$ at low frequency. The E/I ratio [$\frac{G_{i}^{E\to k}\left(t\right)\left(E^{E}-V_{i}^{k}\right)}{G_{i}^{I\to k}\left(t\right)\left(E^{I}-V_{i}^{k}\right)}\text{,}$ see Equation 1] was increased with the reduction of $g^{E\to I}$ (Figure S2C). When C (< 0.22) was small, a slight increase in E/I ratio increased the persistence score, but further increase E/I ratio decreased the persistence score (Figure S2D), suggesting that slightly increased E/I ratio may improve memory recall, but higher E/I becomes detrimental due to overexcitation. Moreover, E/I imbalance increased $S_{p}$ and reduced $S_{l}$ (Figures S2F–S2H), causing difficulty to terminate the cued engram and strong co-activation of memory engrams at low C values (Figure S2E).

### Rescuing memory recall by slow-gamma stimulation

We next studied how reduced connectivity affects the rescue of memory recall by slow-gamma stimulation. In Etter et al. experiment,^14^ optogenetic stimulation at 40 Hz with a 50% duty cycle (on 50% and off 50% of the time), was intended to suppress hippocampal inhibitory neurons, rescued memory recall performance, and restored hippocampal slow-gamma oscillations. To understand the activity dynamics underlying the rescue and the effects of reduced connectivity, we turned on the rescue stimulation in our model (Figure 1D). This simulated optogenetic stimulation by resetting the membrane potentials in 50% of inhibitory neurons to their leakage membrane potentials periodically at a frequency of 10–120 Hz. Except for the first 5 s when the circuit was in a transient state starting from random initial conditions, the stimulations were applied throughout the simulation of memory recall, with a duty cycle of up to 90% on (e.g., a 40% duty cycle would mean 40% on and 60% off). We then investigated how rescue stimulations altered the persistent states associated with the recall of stored memories and related neural oscillations at different C values.

As in previously reported experiments,^14^ we first applied rescue stimulations at a slow-gamma frequency of 40 Hz with a 50% duty cycle and examined the spike raster plots of neurons in multiple memory engrams. For example, at a low C value (0.16), cued memory neurons without rescue stimulations did not always display long-lasting high firing activities after cue input (persistent states), with some terminating early and some missing altogether (Figure 1E top). At the same low C value with rescue stimulations, engrams sometimes entered persistent states spontaneously during background activity, in the absence of the cue input (Figure 1E middle). In response to the cue input when rescue stimulations were present, persistent states emerged in most cued memory neurons (Figure 1E bottom). However, the rescue stimulations also produced something peculiar: a persistent state in one memory engram was triggered by the cue input to another engram, or the persistent state lasted too long and was not reset by the cue input to another engram, resulting in the co-activation of multiple memory engrams (Figure 1E bottom), suggesting the mixed recall of multiple memories.

Using the same metrics of memory neurons described previously, we compared their activities during memory recall (10 s after cue input termination), with and without rescue stimulation. The population mean firing rates increased significantly after rescue stimulation at all levels of C (two-way analysis of variance [2ANOVA], $F_{\left(\begin{array}{cc} 1, & 158 \end{array}\right)}=270.2,p=1.93\times 10^{-33}$, Figure 1F). While the synchrony index increased slightly (2ANOVA, $F_{\left(\begin{array}{cc} 1, & 158 \end{array}\right)}=92.58,p=7.52\times 10^{-17}$, Figure 1G), the proportion of high-firing neurons was significantly higher after rescue stimulation (2ANOVA, $F_{\left(\begin{array}{cc} 1, & 158 \end{array}\right)}=46.19,p=3.36\times 10^{5}$, Figure 1H).

We then compared the metrics of persistent states with and without rescue stimulations. The same threshold of 5 $s^{-1}$ was used to detect persistent states when rescue stimulations were applied, because a bimodal firing rate distribution was also present during memory recall (Figure S1E), with the difference that such a bimodal distribution also occurred when rescue stimulations were applied to background activity in the absence of the cue input (Figure S1F). We found that the persistence score was significantly increased by rescue stimulations (2ANOVA, $F_{\left(\begin{array}{cc} 1, & 78 \end{array}\right)}=75.76$, $p=7.04\times 10^{-15}$, Figure 1I). Therefore, our simulation result confirmed the rescue of memory recall by slow-gamma stimulations reported in previous experiments.^14^ In addition, the overlapping proportion, which was low at all C values without rescue stimulations, increased, especially for the low-*C*-value (≤0.19) regime (2ANOVA, $F_{\left(\begin{array}{cc} 1, & 78 \end{array}\right)}=28.83$, $p=3.26\times 10^{-7}$, Figure 1J). Thus, our simulation showed that rescue stimulations produce a side effect of co-activating multiple memories during the recall of only one memory. By studying bifurcation diagram (relation among f, $f_{M1}$ and C) with rescue stimulations (See STAR Methods for details), we found that the bifurcation curves were shifted to the left (Figure 1L) and hence $S_{p}$ was increased, but $S_{l}$ was reduced (1ANOVA, $F_{\left(6,63\right)}=127.99,p=2.41\times 10^{-33}\text{,}$ Figure 1M blue; 1ANOVA $,F_{\left(6,63\right)}=3.41,p=0.0056\text{,}$ Figure 1N blue). Thus, the persistence score was improved, but overlapping proportion was increased in low-*C*-value, providing a computational explanation of the observations in Figures 1I and 1J blue. Additional analysis showed that reducing the duty cycle of rescue stimulations to 10%–40% increased the persistence score and decreased the overlapping proportion at low C values (Figures S1G and S1H), suggesting better memory rescue with fewer side effects when slow-gamma stimulations were applied at a lower duty cycle. As duty cycle represents the proportion of time inhibitory neurons being suppressed, the result suggests that less suppression to inhibitory neurons improves rescue performance, which may be meaningful in application.

We further analyzed how rescue stimulations alter neural oscillations. In a previous experiment of memory rescue in mice with AD by 40 Hz with 50% duty cycle optogenetic stimulations,^14^ slow-gamma oscillations (30–60 Hz) were enhanced while theta (4–12 Hz) and fast-gamma (60–120 Hz) oscillations were unchanged. Our simulation results were consistent with this experimental finding. When C was less than 0.25 and rescue stimulations were applied, there was a significant enhancement in slow-gamma power (2ANOVA, $F_{\left(\begin{array}{cc} 1 & 158 \end{array}\right)}=319.94,p=6.32\times 10^{-39}\text{,}$ Figure 1P), but not in theta (2ANOVA, $F_{\left(\begin{array}{cc} 1 & 158 \end{array}\right)}=0.64,p=0.43\text{,}$ Figure 1O) or fast-gamma power (2ANOVA, $F_{\left(\begin{array}{cc} 1 & 158 \end{array}\right)}=2.63,p=0.11\text{,}$ Figure 1Q) compared with baseline values.

**Figure 2:**
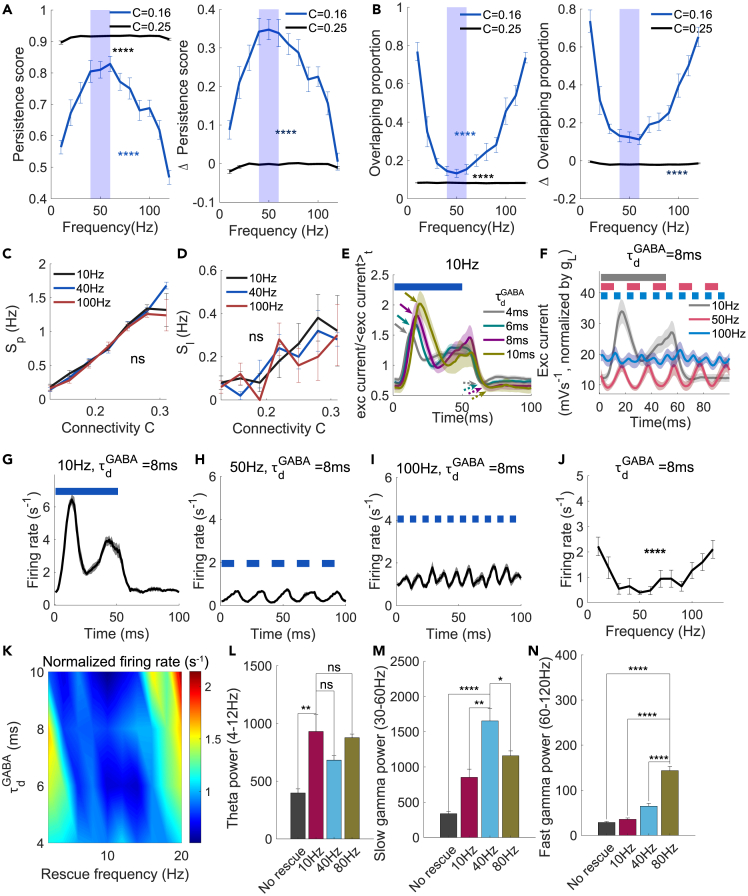
Rescue stimulations are more effective at slow-gamma frequencies (A and B) Persistence score and (B): overlapping proportion and corresponding changes (effect size) after applying different rescue stimulation frequencies in the cases of high (0.25) and low (0.16) C values. The duty cycle was always set at 50%. The shaded area in (A, B) indicates the slow-gamma band. (C and D) $S_{p}$, and (D): $S_{l}$ at different rescue frequencies. (E) Mean normalized excitatory current to non-cued neurons at different $\tau_{d}^{GABA}$ when 10Hz 50% duty cycle rescue stimulations were applied. Solid arrows and dash arrows indicate peak and trough respectively. Broken blue bars indicate the on-off duty cycle. (F) Mean excitatory current when rescue stimulations at different frequencies are applied. Broken bars with corresponding colors indicate the on-off duty cycle, respectively. (G–I) Average population mean firing rate of non-cued neurons during the periods when rescue stimulations are applied. Broken blue bars indicate the on-off duty cycle with different frequencies: 10Hz (G), 50Hz (H), and 100Hz (I). (J) Population means firing rate of non-cued neurons at different frequencies. (K–N) Normalized firing rate at different rescue frequencies and $\tau_{d}^{GABA}$. (L-N): Power of theta (L), slow-gamma (M), and fast-gamma (N) oscillations, with or without rescue stimulations, at different frequencies in the case of a low C value (0.16). Data are represented as mean ± SEM. n = 10 trials/setting, except (K). n = 10 trials/setting. In (A, B), 1ANOVA test; in (C, D) 2ANOVA test; in (L-N), two-sample Student’s *t* test. ns, p > 0.05; ∗, p < 0.05; ∗∗, p < 0.01; ∗∗∗∗, p < 0.0001. See also Figure S2.

We sought to determine whether 40 Hz was the optimal stimulation frequency for memory rescue and how the effects of rescue stimulations on persistent states and neural oscillations depended on the stimulation frequency. We found that at a low C value (0.16), persistence scores showed the greatest improvement when the frequency was in the range of 40–60 Hz (one-way analysis of variance [1ANOVA], comparison between different frequencies, $F_{\left(\begin{array}{cc} 11 & 108 \end{array}\right)}=10.91,p=1.31\times 10^{-12}$,Figure 2A left). The overlapping proportions were the lowest in the same 40–60 Hz frequency range, compared with other frequencies (1ANOVA, $F_{\left(\begin{array}{cc} 11 & 108 \end{array}\right)}=13.62,p=1.01\times 10^{-15}$, Figure 2B left). We noted that at a low C value (0.16), the effects of stimulation frequency on both the persistence score (2ANOVA, $F_{\left(\begin{array}{cc} 1 & 238 \end{array}\right)}=384.88,p=8.82\times 10^{-51}$, Figure 2A right) and the overlapping proportion (2ANOVA, $F_{\left(\begin{array}{cc} 1 & 238 \end{array}\right)}=220.49,p=2.63\times 10^{-35}$, Figure 2B right) were much more prominent. Furthermore, in the study of hysteresis, we found that the improvement in memory performance cannot be simply explained by $S_{p}$ and $S_{l}$ of the cued memory. We observed that $S_{p}$ and $S_{l}$ were similar for different frequencies of the rescue stimulation (2ANOVA, $F_{\left(\begin{array}{cc} 2 & 207 \end{array}\right)}=1.28,p=0.28$,Figure 2C; 2ANOVA, $F_{\left(\begin{array}{cc} 2 & 207 \end{array}\right)}=0.42,p=0.66$, Figure 2D), suggesting that the activation of the cued neurons is insensitive to different frequencies, which seems contradictory with the previous observation that the memory performance is sensitive to frequency (Figures 2A and 2B). To understand the cause of contradiction, we further analyzed the non-cued neurons. The result showed that oscillation provided additional advantage in memory performance. The mechanism is that during the off-to-on and on-to-off periods, the excitatory current needs proper time to rise to the peak and fall to the trough, respectively (Figure 2E). The optimal rescue frequency should provide enough time for the excitatory current to decay in the off period but not too long for non-cued neurons to accumulate membrane potential in the on period in order not to induce too strong excitatory current and firing of these non-cued neurons (Figure 2F). If the rescue frequency was too low, the non-cued neurons would accumulate membrane potential in the on period, and the population mean firing rate, thus increased (Figure 2G). If the rescue frequency was too high, the excitatory current would maintain at a high value, because of not enough time to decay in the off period, and the populations mean firing rate did not respond to rescue stimulations and stayed at high value (Figure 2I). As slow-gamma stimulations at 40–60 Hz provided suitable on-off period to induce minimal excitatory current to the non-cued memory neurons, thus reduced the firing rate (Figure 2H), while persistent state could still be maintained in the cued memory neurons. Slow-gamma stimulations generated less firing of non-cued neurons (1ANOVA, $F_{\left(\begin{array}{cc} 11 & 108 \end{array}\right)}=4.26,p=2.95\times 10^{-5}$, Figure 2J) and provide additional suppression to non-cued neurons. In this way, memory can be cued without inducing too strong overlapping with other non-cued memories in the presence of suitable rescue frequency. Furthermore, the raising time to the peak and falling time to the trough was increased with $\tau_{d}^{GABA}$ (Figure 2E). Therefore, the optimal rescue frequency should reduce to match the longer time to reach peak and trough when $\tau_{d}^{GABA}$ increases (Figure 2K).

For neural oscillations, we found that theta power was enhanced by rescue stimulations at a frequency of approximately 10 Hz, but was not significantly different from that by stimulation frequency in slow- and fast-gamma range (Figure 2L), but slow- or fast-gamma power was most effectively enhanced by stimulations at the same slow- or fast-gamma frequency (Figures 2M and 2N), as expected from previous experimental results.^14^ Overall, our results suggest that stimulations at the slow-gamma band are more effective for memory rescue than those at other frequencies.

Our design of simulation was based on Etter et al. experiment^14^ and we found that the effect of rescue stimulations is partially due to disinhibition of the excitatory neurons. Therefore, we wondered if general increase of excitability can also rescue memories. We tested the effect by providing stronger background input $f_{background}^{exc}$ to excitatory neurons, while the background input to inhibitory neurons was maintained at $f_{background}=2.5Hz$. We found that increasing excitability partially improved persistent state metrics at a low value of *C* (Figures S2M–S2R), suggesting that excitability could be manipulated for memory rescue.

### Connectivity reduction impairs learning a new memory in the presence of existing memories

**Figure 3:**
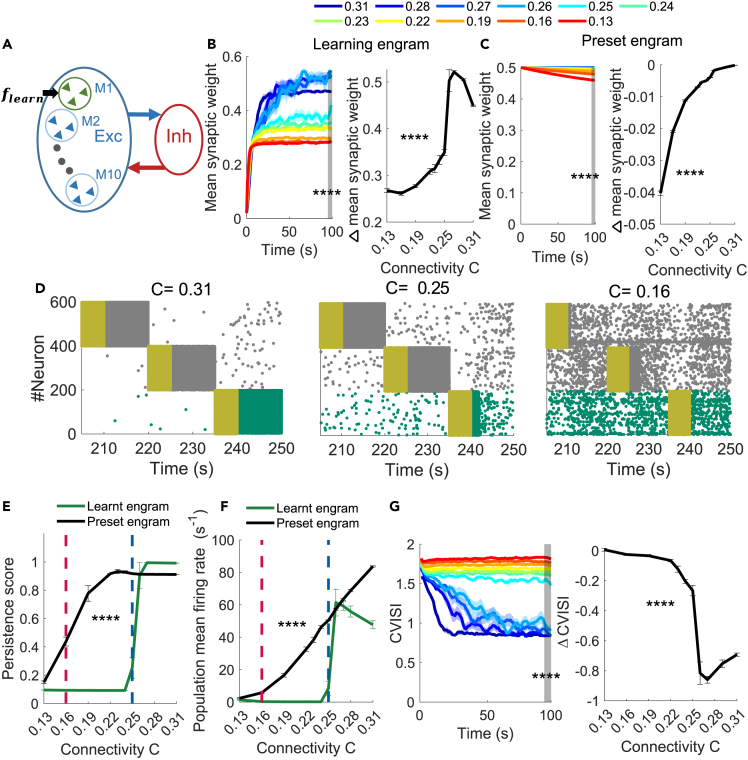
Low connectivity impairs the learning of new memory engrams (A) Learning model with nine preset memory engrams (M2–M10) and a learning engram (M1) that is left for learning by receiving a learning signal $f_{learn}$. (B and C) Evolution (left) and changes between 100 s and 1 s (right) of the mean synaptic weight within the learning engram (B) and preset engrams (C) during learning with different connectivities, C. (D) Spike raster plot of the learnt and two preset engrams during recall at different C values (C = 0.31, 0.25, 0.16). Gray/green: spikes of preset/learnt engrams, yellow: cue input. (E and F) Persistence score (E) and population mean firing rate (F) of the learnt engram (green) and preset engrams (black) at different C values during recall after learning. Vertical red and blue dashed lines indicate C = 0.16 and C = 0.25, respectively. (G) Evolution (left) and changes between 100 s and 1 s (right) of the coefficient of variation of the inter-spike interval (CVISI) within the learning engram during learning with different C values. Data in (B, C, E, F, G) are represented as mean ± SEM. n = 10 trials/setting. ∗∗∗∗, p < 0.0001 in 2ANOVA test; (E, F), the value at 100 s in 1ANOVA test (gray region in B, C, G). See also Figures S2 and S3.

In the work described in the previous sections, memory engrams were preset in the model to study memory recall and rescue. Here, we focused on how reduced C affects the learning of a new memory in the presence of preset memories. To this end, we considered a learning model (Figure 3A), consisting of nine preset memory engrams (neuron number 200–1,999) and one learning engram (neuron number 0–199) driven by a learning signal, which was a strong external input (12.5 Hz) applied for 100 s to the learning engram. The synaptic weights between neurons in the learning engram began at an initial non-coding value. When the learning signal was present, all excitatory synapses between all excitatory neurons (including those in the preset engrams) were dynamically changed according to a learning rule (see STAR Methods for details).^38^^,^^39^^,^^40^^,^^41^^,^^42^^,^^43^ Afterward, learning was terminated (synaptic weights were fixed) and all preset and newly learnt memory engrams were sequentially recalled by applying the cue input one by one, as in our previous model (Figure 1A). We analyzed how C affected learning-induced changes in the synaptic weights and recall activities of the newly learnt and preset engrams.

We first examined how the synaptic weight evolves in the learning and preset engrams during learning depended on C. When C was large (> 0.25), the synapses between neurons targeted for learning tended to potentiate to a large value and form memory engrams, while when C was small (< 0.25), they tended to potentiate much less (1ANOVA comparing values at *t* = 100 s among different *C* values, $F_{\left(\begin{array}{cc} 1 & 108 \end{array}\right)}=134.77,p=5.11\times 10^{-53}$, Figure 3B left; 1ANOVA, $F_{\left(\begin{array}{cc} 1 & 108 \end{array}\right)}=81.11,p=5.69\times 10^{-47}$, Figure 3B right). In the preset engrams, the synapses were not affected by learning the new memory at large C values (> 0.25), and more synapses tended to depress as C was reduced, but the synaptic weight reduction was modest (1ANOVA test at t = 100s, $F_{\left(\begin{array}{cc} 1 & 108 \end{array}\right)}=113.45,p=4.76\times 10^{-54}$, Figure 3C left; 1ANOVA test, $F_{\left(\begin{array}{cc} 1 & 108 \end{array}\right)}=2352,p=0.00$, Figure 3C right).

We then examined the memory recall of the learnt engram after learning at different C values. Examples of spike raster plots during recall showed that persistent states were successfully activated at a high C value (0.31) for the learnt engram (Figure 3D, left), whereas at small C values, they were either truncated or not activated (Figure 3D, middle and right). Our quantifications confirmed this observation. The persistence score and population mean firing rate of the learnt engram were high at large C values (> 0.25; Figures 3E and 3F). Thus, in this regime, the new memory was successfully encoded. As C values were reduced (< 0.25), the persistence score and firing rate of the learnt engram decreased to almost zero, indicating impairment in the encoding of the new memory. This encoding impairment was most likely caused by the low synaptic weights after learning at low *C* values (Figure 3B) because further analysis showed that synaptic strength within a memory engram needs to be sufficiently strong for a cue input to produce persistent states during memory recall (Figures S2I–S2L). In addition, we formally compared these measurements between the learnt and preset engrams during memory recall. Both measures were significantly lower for the learnt engram than the preset engrams, especially at low *C* values (persistence score: 2ANOVA, $F_{\left(\begin{array}{cc} 1 & 238 \end{array}\right)}=58.81,p=2.86\times 10^{-28}$, Figure 3E; population mean firing rate: 2ANOVA, $F_{\left(\begin{array}{cc} 1 & 238 \end{array}\right)}=39.36,p=1.57\times 10^{-9}\text{,}$ Figure 3F). Our analysis thus indicates that low connectivity impairs the encoding of the learning engram, such that it cannot be properly recalled later as a preset engram.

We performed additional analysis to understand how the encoding impairment in the learning engram at low C values was produced during learning through the plasticity rule in our model. One clue came from our observation that at high *C* values, but not at low *C* values*,* neurons in the learning engram changed their firing burstiness during learning (Figure 3G), as quantified by the coefficient of variation of the inter-spike interval (CVISI), with a value of 1 indicating Poisson firing and a value > 1 indicating burst firing. At the beginning of learning, neurons in the learning engram were bursty (CVISI > 1.55) across all *C* values (driven by the learning signal). At the end of learning, however, they became much less bursty (CVISI close to 1) at high *C* values (> 0.25), but remained bursty at low *C* values (< 0.25) (1ANOVA, $F_{\left(\begin{array}{cc} 1 & 118 \end{array}\right)}=103.84,p=3.69\times 10^{-52}$; Figure 3G right gray region). Because synaptic weights within the learning engram also showed different dynamic changes at different *C* values during learning (Figure 3B), we probed a possible link between synaptic weights, firing rate, and CVISI in a simple two-neuron model, consisting of a presynaptic neuron and a postsynaptic neuron connected by a plastic synapse (see STAR Methods for details). We found that after reaching a relatively high external firing rate during learning, strong burst firing (CVISI > 1.55) recruited heterosynaptic plasticity in the learning rule in our model, which restricted synaptic potentiation (Figures S3A–S3C). Therefore, the sustained high-burst firing during learning at low *C* values presumably limited the synaptic potentiation within the learnt engram, leading to low synaptic weights and encoding impairment.

We finally examined the recall of preset engrams after learning. The preset engrams were generally recalled with higher persistence scores and population mean firing rates than the learnt engram (Figures 3E and 3F), as described previously. In addition, the recall performances did not significantly differ from those without learning (Figures S3D and S3E). Therefore, the modest weakening of synaptic weights within the preset engrams by learning (Figure 3C) did not further impair the recall of preset engrams, in addition to the impairment caused by low *C*.

Overall, our results showed that at a high level of connectivity (C > 0.25), a new memory engram can be learnt, whereas with reduced connectivity (C < 0.25), the encoding of the new memory engram was impaired. However, the recall of preset memory engrams was largely unaffected by learning.

## Discussion

In this work, we studied the effects of synaptic impairment on the recall and learning processes in a neural network under different levels of connectivity. We first discovered that low C led to changes in the persistent state related to memory recall. Gradually reducing C reduced firing synchrony, but interestingly, increased then decreased the overall population mean firing rate of memory neurons. Moreover, reducing the number of neurons and connection strength induced severer memory impairment because of inducing more damage to the memory engrams, and the increase of E/I ratio found in experiment^12^ might be a remedy to memory performance caused by synaptic impairment. Second, we found that deactivating inhibitory neurons in the slow-gamma frequency effectively enhanced the firing rate and synchrony of cued memory engrams to partially rescue memory recall from engram activation impairment in low-C situations, which is not simply due to the effect of increased excitability by disinhibition. However, low C may lead to the simultaneous activation of multiple memory engrams by slow-gamma rescue stimulations. Third, we studied the learning of a new memory engram in the presence of existing memory engrams. We found that with sufficiently high connectivity, the new memory engram can be successfully learnt. There was a mild reduction regime with impairment in encoding a new memory engram, but the activation of preset engrams was not affected by learning. There have been several simulation studies on the effect of synaptic loss. Abuhassan et al. studied the process of compensation to increase the excitability of neurons and reproduced the shift of the oscillation band to a lower frequency with synaptic loss.^44^^,^^45^^,^^46^ In Armando Romani, et al. simulation,^47^ the increased neurotransmitter release caused by Aβ^48^ was found to cause an increase in spiking probability, but a reduction in synaptic current generated by sequential spiking at different frequencies. In Willem de Haan, et al. work,^49^ different therapeutic strategies were studied using a neural mass model with degenerated human network topology, and it was found that, although increasing the degree of inhibition by inhibitory neurons had favorable effects, the best strategy was to enhance the excitability of excitatory neurons. These studies have focused on the spontaneous dynamics of circuits with synaptic loss, but have not considered the existence and activation of engrams, which are important in learning and memory;^15^^,^^16^ thus, our model has the advantage of filling this gap.

### Relationship between connectivity reduction and memory deficits

From a mechanistic point of view, memory deficits may be caused by a failure to form (encoding impairment) or activate (engram activation impairment) memory engrams. The results of previous experiments^15^^,^^16^ support an engram activation impairment underlying recall impairment in AD.^15^^,^^16^^,^^23^ In Stopford, C.L., et al. Experiment,^23^ some AD patients showed poorer performance at recalling visual and verbal information immediately after a presentation and after a 30-min delay. In this regard, our study shows that severe connectivity deficits induce recall difficulty in activating persistent states associated with previously encoded memory engrams. This is consistent with previous studies showing that persistent states are related to a bifurcation property^31^^,^^33^^,^^50^ induced by changes in local synaptic weight or connectivity. Here, we study the effect of connectivity on the bifurcation property and show that the stability of persistent state reduces when connectivity reduces, causing difficulty in maintaining the persistent state. Experimental studies have shown that compared with apolipoprotein E (APOE) ε4 non-carriers, APOE ε4 carriers have higher brain activity in the early stage of life,^51^^,^^52^^,^^53^ but the old pathological patient in the later stage of disease is characterized by lower brain activity.^53^^,^^54^ Combined with previous findings^2^^,^^55^^,^^56^ that AD patients have lower synaptic connectivity between neurons, we speculated that such a connectivity deficit (reduction in C) would first increase firing and then reduce firing as C further decreases. This speculation is supported by our model simulation results (Figure 1F) and is also consistent with the results of a recent study^57^ showing that densely connected modular networks, compared with sparsely connected networks, have a lower population mean firing rate within modules through increased firing synchrony. The low population mean firing rate, but sufficient excitability, of circuits with sufficient density helps support information processing ability in a normal, healthy primate brain, as a low firing rate is beneficial for metabolic energy saving and may reduce the risk of Aβ deposition, which is thought to be a by-product of metabolism waste.^4^^,^^37^^,^^58^

### Mechanism of memory rescue by suppressing inhibitory neurons

There are reports that memory deficits may be rescued by several measures. Several experimental studies have shown that manipulations through chemical or optogenetic stimulation rescue spatial memory performance.^13^^,^^14^^,^^15^^,^^59^ However, the mechanism underlying memory rescue is elusive. Based on the results of a previous experiment^14^ in which spatial memory performance was rescued by applying optogenetic stimulations at a slow-gamma frequency to medial septum Parvalbumin (PV) cells, we applied slow-gamma frequency stimulations to inhibitory neurons in our model and provided new insights into the mechanism of rescuing previously encoded memories. We revealed that the suppression of inhibitory neurons shifts the hysteresis of bifurcation curve to lower cue input, thus the stability of persistent state is increased. However, the suppression also reduced the stability of low activity state. Thus, memory recall was partially rescued, but the overlapping proportion during memory recall was increased. Other than the suppression of inhibitory neurons, the oscillation induced additionally effect on rescue. The optimal stimulation frequency was around 40 Hz, as this frequency most effectively avoided the over-activation of non-cued excitatory neurons in low C situations by providing suitable period for excitatory current to the non-cued neurons to be minimal, thus better preventing non-cued memory engrams from being activated spontaneously. Even so, we found that the rescue induced an increase in the overlapping activation of memory engrams as a side effect. This phenomenon has not previously been found experimentally and may be worth testing in future experimental studies.

Impairments in the formation of new memories during learning have been reported in AD.^19^^,^^20^^,^^21^^,^^22^^,^^23^^,^^24^ To study how synaptic impairment affects learning impairment, our model used a composited plasticity rule, including spike-timing-dependent plasticity (STDP), heterosynaptic plasticity, and transmitter-induced plasticity, to study the engram formation ability under conditions of connectivity deficit. These plasticity forms were identified in previous experiments^39^^,^^41^^,^^42^^,^^43^^,^^60^^,^^61^^,^^62^^,^^63^^,^^64^ are biologically plausible. The ability to form engrams using STDP has been known for a long time.^39^^,^^60^^,^^65^ However, the traditional STDP rule suffers from explosive synaptic weight potentiation.^61^^,^^62^^,^^63^^,^^64^ To prevent an explosion, different modification forms of STDP rules^66^^,^^67^^,^^68^ or additional plasticity rules^39^^,^^69^ were used. Among above, we used heterosynaptic plasticity, as previously described.^39^ Previous experiments have shown that heterosynaptic plasticity is activated in response to a high neuronal firing rate^42^^,^^43^ to restrain synaptic potentiation. Therefore, this plasticity rule ensures that the synaptic weight does not explode at any neuronal firing rate. We also used transmitter-induced plasticity^39^^,^^41^ in our study to prevent neurons from becoming silent during the simulation. Our model incorporating these biologically plausible plasticity rules suggested that mild connectivity deficits affect the formation of new engrams first, but do not strongly affect existing memory engrams. This may reflect the mechanism of new memory impairment found in AD patients.^19^^,^^20^^,^^21^^,^^22^^,^^23^^,^^24^ With a severe reduction of connectivity, the preset memory engrams cannot be activated after learning a new memory, which is consistent with the loss of long-term memory in AD patients.^15^^,^^22^^,^^23^^,^^24^

The reduced ability to form new engrams is related to the increase in burst firing at low levels of connectivity, which induces stronger heterosynaptic plasticity to restrain the increase in synaptic weight while learning the new engram. This model prediction of the mechanism underlying impairments in learning new memories may need to be further examined in future experiments.

### Limitations of the study

Furthermore, various types of plasticity and intrinsic property of neurons are affected by neural impairment. Homeostatic plasticity has been found to be abnormal in AD patients.^70^^,^^71^ Moreover, A β concentration has been found to affect the activation of *N*-methyl-d-aspartate (NMDA) receptors,^3^^,^^8^ which is crucial for STDP.^72^^,^^73^^,^^74^ The activation of NMDA receptors in AD patients is potentiated in the early stage of disease and reduced in the late stage of disease.^3^ Abnormal NMDA receptor activation in AD would, thus, lead to impaired plasticity. Lastly, the excitability of neurons reduces with aging and AD.^75^^,^^76^ AD plaque was found to reduce inhibition of nearby synapses.^77^ The effect of homeostatic plasticity and impaired plasticity itself are not considered in our model and warrant inclusion in further studies of connectivity deficits.

## STAR★Methods

### Key resources table

**Table:** 

REAGENT or RESOURCE	SOURCE	IDENTIFIER
**Software and algorithms**		

MATLAB	MathWorks	https://www.mathworks.com/products/matlab/
Statistical Toolbox	MathWorks	https://www.mathworks.com/products/statistics.html
gcc complier	GNU project	https://gcc.gnu.org/
MT19937	Yukihiro Matsumoto	http://www.math.keio.ac.jp/∼matumoto/emt.html
Code archived	This manuscript	https://doi.org/10.5281/zenodo.7107136

### Resource availability

#### Lead contact

Further information and requests for resources and reagents should be directed to and will be fulfilled by the Lead Contact, Changsong Zhou (cszhou@hkbu.edu.hk).

#### Materials availability

All unique resources generated in this study are available upon request.

### Method details

#### Neural circuit model

##### Circuit architecture and dynamics

The model circuit we used was composed of 2,400 neurons, with 2,000 excitatory (E), $n_{E}\text{,}$ and 400 inhibitory (I), $n_{I}$, neurons (Figure 1A). Neurons were connected randomly with directed synapses with various probabilities, C. Four hundred independent Poisson trains with a rate of $f_{background}=2.5\;Hz$ were applied to each neuron to mimic the input from other neural circuits. The neural dynamics in the network were modeled by integrate-and-fire neurons with α-amino-3-hydroxy-5-methyl-4-isoxazolepropionic (AMPA)-, N-methyl-D-aspartic acid receptor (NMDA)-, and gamma-aminobutyric acid (GABA)-receptor-mediated conductance^25^ as follows:

(Equation 1) $$\tau^{k}\frac{{dV}_{i}^{k}}{dt}=V_{L}-V_{i}^{k}+G_{i}^{E\to k}\left(t\right)\left(E^{E}-V_{i}^{k}\right)+G_{i}^{I\to k}\left(t\right)\left(E^{I}-V_{i}^{k}\right)$$

(Equation 2) $$G_{i}^{I\to k}\left(t\right)=\tau^{k}\sum_{j\in \partial_{i}^{I}}\sum_{n}w_{j\to i}S^{GABA}\left(t-t_{j}^{n}\right)$$

(Equation 3) $$G_{i}^{E\to k}\left(t\right)=\tau^{k}\left[\sum_{j\in \partial_{i}^{O}}\sum_{n}g^{O\to k}S^{AMPA}\left(t-t_{j}^{n}\right)+\sum_{j\in \partial_{i}^{E}}\sum_{n}w_{j\to i}u_{j}\left(t\right)x_{j}\left(t\right)\left\{S^{AMPA}\left(t-t_{j}^{n}\right)+S^{NMDA}\left(t-t_{j}^{n}\right)\right\}\right]\text{.}$$

Here, k=EorI denotes the neuron type and $\partial_{i}^{k}$ indicates the neighbors with k neuron type of neuron i. O→k indicates the external input to neuron type k. $V_{i}^{k}\left(t\right)$ is the membrane potential of neuron i and neuron type k, and $t_{j}^{n}$ is the *n*-th spike of neuron j. The synaptic time course is described by the following bi-exponential function:

(Equation 4) $$S^{R}\left(t\right)=\mu^{R}\frac{\Theta \left(t-\tau_{l}\right)}{\tau_{d}^{R}-\tau_{r}^{R}}\left(e^{-\frac{t-\tau_{l}}{\tau_{d}^{R}}}-e^{-\frac{t-\tau_{l}}{\tau_{r}^{R}}}\right)\text{,}$$

where $\tau_{r}^{R}$ and $\tau_{d}^{R}$ are the characteristic rising time constant and decay time constant, respectively, and $\mu^{R}$ is the amplitude, depending on the receptor type R (AMPA, GABA, or NMDA) of the presynaptic neurons. Θ(t) is the Heaviside step function. $w_{j\to i}$ is the synaptic weight from the presynaptic neuron, j, to the postsynaptic neuron, i, and was initially set as

(Equation 5) $$w_{j\to i}=\left\{ \begin{array}{c} g^{E\to E},i\in E,j\in E\\ g^{I\to E},i\in E,j\in I\\ g^{E\to I},i\in I,j\in E\\ g^{I\to I},i\in I,j\in I \end{array}\right.\text{,}$$

Two hundred excitatory neurons were randomly assigned to an engram representing a memory and there were several non-overlapping memory engrams considered in our model. The synaptic weights within each engram were replaced from the baseline value, $g^{E\to E}$, to a larger value, $g_{M}^{E\to E}$.

##### Short-term plasticity

Presynaptic short-term plasticity was described by the dynamics of two variables, the amount of neurotransmitter $,x_{i}$, and the release probability $,u_{i}$, in Equation 3, and was governed by a depression time constant $,\tau_{D}$, and a facilitation time constant, $\tau_{F}$, as described in the following equations ($\tau_{F}\gg \tau_{D}$):

(Equation 6) $$\frac{{du}_{i}\left(t\right)}{dt}=\frac{U-u_{i}\left(t\right)}{\tau_{F}}+U\left(1-u_{i}\left(t\right)\right)S_{i}\left(t\right)$$

(Equation 7) $$\frac{{dx}_{i}\left(t\right)}{dt}=\frac{1-x_{i}\left(t\right)}{\tau_{D}}-u_{i}\left(t\right)x_{i}\left(t\right)S_{i}\left(t\right)\text{,}$$

where $S_{i}\left(t\right)=\sum_{n}\delta \left(t-t_{i}^{n}\right)$ is the spike train of neuron iandδ(t) is the Dirac delta function. Depending on the firing history of a neuron, short-term depression or potentiation^31^^,^^78^^,^^79^ may be induced by the depletion of neurotransmitter, $x_{i}$, or an increase in the release probability, $u_{i}$, which temporarily changes the synaptic conductance, $G_{i}^{E\to k}\left(t\right)$, in Equation 3.

##### Long-term plasticity

The long-term change in synaptic weight obeys an integrative plasticity rule, which includes triplet STDP, heterosynaptic plasticity, and transmitter-induced plasticity.^39^ The plasticity rule was applied to all E-to-E synapses. The synaptic weight evolved according to the following formula:

(Equation 8) $$\frac{dw_{j\to i}}{dt}=\underset{Triplet\;STDP}{\underset{︸}{S_{i}\left(t\right)\cdot \left(Az_{j}z_{i}^{slow}\right)-S_{j}\left(t\right)\cdot \left(Bz_{i}\right)}}-\underset{Heterosynaptic\;plasticity}{\underset{︸}{S_{i}\left(t\right)\cdot \beta z_{i}^{3}{\left(\frac{w_{j\to i}-\tilde{w}}{\tilde{w}}\right)}^{3}}}+\underset{\begin{array}{c} Transmitter\\ induced\;plasticity \end{array}}{\underset{︸}{\delta_{1}S_{j}\left(t\right)}}\text{,}$$

where j→i indicates the connection from neuron j to neuron i. The synaptic traces evolved as follows:

(Equation 9) $$\frac{{dz}_{i}}{dt}=-\frac{z_{i}}{\tau_{STDP}}+S_{i}\left(t\right)$$

(Equation 10) $$\frac{{dz}_{i}^{slow}}{dt}=-\frac{z_{i}^{slow}}{\tau_{STDP_{slow}}}+S_{i}\left(t\right)\text{.}$$

Synaptic weight modification was controlled by the presynaptic trace, $z_{j}\text{,}$ and the postsynaptic traces, $z_{i}$ and $z_{i}^{slow}$, which increased by one with every spike and decayed to zero with the characteristic time constants, $\tau_{STDP}$ and $\tau_{STDP\_slow}$, respectively. The slow synaptic trace, $z_{i}^{slow}\text{,}$ kept the spiking information of the long timescale $\tau_{STDP\_slow}$, which made the synaptic potentiation term of the triplet STDP, $S_{i}\left(t\right)\cdot \left(Az_{j}z_{i}^{slow}\right)\text{,}$ depends on the postsynaptic spiking rate and increased the synaptic weight by the amount $Az_{j}z_{i}^{slow}$ with every spike. The depression term of the triplet STDP, $-S_{j}\left(t\right)\cdot \left(Bz_{i}\right)$, reduced the synaptic weight by the amount $Bz_{i}$ with every presynaptic spike. The triplet STDP rule alone was unstable because potentiated (depressed) synapses tend to induce more (less) postsynaptic spikes, which in turn potentiate (depress) the synapses. To stabilize synaptic weight, heterosynaptic plasticity, the third term in Equation 8, was introduced in Zenke et al.^39^ Heterosynaptic plasticity reduces the synaptic weight by the amount $\beta z_{i}^{3}{\left(\frac{w_{j\to i}-\tilde{w}}{\tilde{w}}\right)}^{3}$, where $\tilde{w}$ is the preferred synaptic weight at every postsynaptic spike. As the growth of heterosynaptic plasticity is in the fourth order ($z_{i}^{3}S_{i}\left(t\right)$), which is higher than the third order ($S_{i}\left(t\right)z_{j}z_{i}^{slow}$) in triplet STDP, the growth of heterosynaptic plasticity is faster than triplet STDP and able to suppress the synaptic weight explosion caused by instability of triplet STDP. Heterosynaptic plasticity has been identified in hippocampus, visual and auditory cortex^42^^,^^43^ by postsynaptic stimulation and is believed to occur in the high-firing-rate domain to prevent the synaptic weight from running away.^42^^,^^43^ Therefore, we modeled heterosynaptic plasticity as the third power of the postsynaptic trace, $z_{i}^{3}$. Transmitter-induced plasticity, the last term in Equation 8, prevents neurons from being silent, reflecting spontaneous spine growth^41^ or a potentiation mechanism that counteracts Hebbian long-term depression.^40^ In addition, according to Dale’s principle, a lower bound of 0.001 was set for each plastic synapse to prevent negative and zero weights. The parameters of the model and the plasticity rules are shown in Table S1*.*

#### Simulation of recall, rescue, and learning

From the beginning of the simulation, each neuron received 400 independent Poisson pulse trains at 2.5 Hz as the background external input to mimic the inputs from other neural circuits, which were present during the entire simulation time. The simulation was first run for 5 s to avoid a transient state. When studying memory recall, memory engrams were activated in sequence by adding an additional external cue input of 10 Hz, $f_{cue}$, to one engram for 5 s. The next memory engrams were then cued consecutively after 10 s (Figure 1B bottom). Therefore, the total time interval between the start of the cue of consecutive memory engrams was 15 s.

To study memory rescue, rescue stimulations, with a duty cycle of 10%–90%, were applied to 50% of the inhibitory neurons randomly selected at the beginning of the simulation, at a frequency of 10–120 Hz. A duty cycle of 10%–90% corresponds to on-stimulus for the first 10%–90% of time in each cycle of the stimulation and off-stimulus for the remaining time. When the stimulus in the rescue stimulations was on, the membrane potential of each chosen inhibitory neuron, if it was not within the refractory period, had a 50% chance per time step to be reset to its leakage potential, $V_{L}$, which prevented the chosen neurons from firing at the moment of applying the rescue stimulation.

When studying the learning of a new memory in addition to existing memories at different Cvalues, nine engrams were preset and one additional engram (neuron number 0–199) was left for learning (learnt engram). The synaptic weights between neurons in the learning engram started at an initial non-coding value of *g*^*E→E*^ = 0.02. After a 5 s period of simulation with the background input, the learning process began and lasted for 100 s, during which Poisson learning inputs at 12.5 Hz were applied to the learnt engram in addition to the background input. In the learning period, all synaptic weights between the excitatory neurons, including those in the preset engrams, were updated according to the learning rule described above (Equation 8. We tuned the learning rule parameters such that the distribution peak of the synaptic weight after learning was located at approximately 0.5–0.6 for high C values (Figure 3B), which is comparable to the average, $g_{M}^{E\to E}$, within the preset engrams. After learning, changes in synaptic weight were prohibited and all preset and new memory engrams were cued one by one for memory recall.

#### Bifurcation analysis of engram

To study the relationship between persistent state and C, we kept only one engram, M1 and started our simulation by applying background input with frequency $f_{background}$ to all neurons, except neurons in M1 which received cue input with frequency $f_{M1}$. $f_{M1}$ is increased by 0.2Hz every 1s until ${f_{M1}=f}_{background}+2$ Hz, then $f_{M1}$ is reduced by 0.2Hz every 1s until ${f_{M1}=f}_{background}-2$ Hz. Population mean firing rate of M1, f, is calculated every second to figure out the relation between $f_{M1}$ and f. For the condition of having rescue stimulation, 40 Hz 50% duty cycle pulses were applied as described in simulation of recall, rescue, and learning*.*

If there is no intersection point larger than $f=5s^{-1}$ at $f_{M1}=f_{background}$ in Figures 1K and 1L, persistent state does not exist for the circuit with background input $f_{background}$. When M1 undergoes state change, $f_{M1}$ has to go through the bifurcation point $\max\left[f_{M1}\left(f=5s^{-1}\right)\right]$ for switching from low activity state to persistent state; while $f_{M1}$ has to go through another bifurcation point $\min\left[f_{M1}\left(f=5s^{-1}\right)\right]$ for switching from persistent state to low activity state. We thus defined two quantities $S_{p}$ and $S_{l}$, as $f_{background}-\min\left[f_{M1}\left(f=5s^{-1}\right)\right]$ and $\max\left[f_{M1}\left(f=5s^{-1}\right)\right]$, to represent the stability of persistent state and low activity state respectively.

#### Simple model of two connected neurons

To gain insights into how synaptic weights change with different presynaptic and postsynaptic firing rate and firing pattern combinations, we built a simple two-neuron model with one synapse to learn using the learning rule described in Equation 8. The parameters used were the same as those listed in Table S1. The simulation lasted for 100 s and the firing activities of the two neurons were generated from a Poisson random process with fixed rates that did not change with learning. Therefore, in this model, the causality of presynaptic firing increasing the firing of postsynaptic neurons and the effect of synaptic weight change during learning on firing dynamics were ignored.

In addition to firing rate, the bursty firing of neurons, which was represented by the CVISI (higher CVISI, more bursty), also affected the synaptic weight. To study the effect of the CVISI on synaptic weight change (under a fixed total number of spikes), the spike trains of the presynaptic and postsynaptic neurons used in the two-neuron model were manipulated to introduce burst firing. N spikes were randomly chosen with a probability $p_{pick}$, and their corresponding spike times were $t_{pick}^{n},n=1,2,\ldots N$. Two new spikes were added after every picked spike, at $t_{pick}^{n}+2ms$ and $t_{pick}^{n}+4ms$, to form a new spike train. 2 N spikes were then removed randomly from the new spike train to maintain the same number of spikes (N spikes; see illustration in Figure S3B). Under this manipulation, the CVISI increased when $p_{pick}$ increased, as more spikes were reorganized to turn into the burst firing of 2–3 spikes.

#### Simulation details

All simulation programs were written in C++ and run on a computer cluster supported by the High-Performance Cluster Computer Center at Hong Kong Baptist University. The equations for the circuit model and plasticity rules were simulated using the second-order Runge–Kutta method, with a time step of 0.05 ms and the spike time correction method.^80^ The parameters used in simulations were referred to and modified from published articles.^25^^,^^31^^,^^39^ The parameters of conductance-based E-I neuronal circuit model can be referred to Yang et al. study^25^ about cost-efficiency of E-I neuronal circuit (Detailed information at circuit architecture and dynamics). The parameters of short-term plasticity model can be referred to Mongillo et al. study^31^ about the neurotransmitter dynamics at synapse (Detailed information at short-term plasticity). The parameters of long-term plasticity model (triplet STDP, heterosynaptic plasticity, transmitter-induced plasticity) can be referred to Zenke et al. study^39^ about learning and memory formation (Detailed information at long-term plasticity). The parameters of the model and the plasticity rules from these references are shown in Table S1*.*

#### Statistical index and analysis

##### Firing properties

We quantified the firing properties of neurons in cued-memory engrams (memory neurons) and outside the engrams (non-cued memory neurons). When studying the persistent state, we computed the population mean firing rate, which is the number of spikes fired by all neurons in an engram in a time window of 10 s from the end of their cue input, divided by the window time and the number of neurons in the engram. Moreover, when studying the learning process, we computed the firing rate distribution for the last 20 s of learning.

In addition, we computed the proportion of high-firing neurons in the engram, defined as those with an individual firing rate larger than 5 $s^{-1}$, which is the firing threshold separating the low-activity state from the persistent state, as described in results, from the cue input termination to 10 s after termination.

To quantify the synchronization of neurons, the synchrony index, $K_{ij}$, which is a measure of the probability of a pair of neurons firing coincidently, was defined as,

$$K_{ij}=\frac{\sum_{k=1}^{l}B_{i}\left(k\right)B_{j}\left(k\right)}{\sqrt{\sum_{k=1}^{l}B_{i}\left(k\right)\sum_{k=1}^{l}B_{j}\left(k\right)}}\text{,}$$

where $B_{i}\left(k\right)$ and $B_{j}\left(k\right)$ indicate neuron i and j, respectively, with a spike at the *k*-th 1 ms bin (0 for no spike and 1 for having a spike). A high synchrony index indicates that the neurons tend to fire coincidently.

In the study of the effect of $\tau_{d}^{GABA}$ and rescue frequency, we used normalized firing rate to compare the firing rates of non-cued neurons while applying different rescue stimulation frequencies and particular synaptic decay time constant of GABA ($\tau_{d}^{GABA}$). Normalized firing rate was calculated as below:

$$Normalized\;firing\;rate\left(Rescue\;frequency,\tau_{d}^{GABA}=a\right)=\frac{Firing\;rate\;\left(Rescue\;frequency,a\right)}{mean\left(Firing\;rate\;\left(Rescue\;frequency,a\right)\right)}$$

##### Persistent states

To study memory recall, we detected cue-induced persistent states for each memory engram. A persistent state of an engram is defined as the period with a population mean firing rate larger than 5 $s^{-1}$, to separate the persistent state from a low-activity state, which is also valid when implementing rescue stimulations, detected using a moving window of 1 s with a time step of 1 ms. If a gap with a low population mean firing rate (<5 $s^{-1}$) lasting for less than 1s appeared between two persistent states, the gap was ignored and the two persistent states were counted as one persistent state.

The firing rate threshold was determined from the firing rate distribution of all excitatory neurons under different conditions (Figures S1C–S1F). It was calculated using a 1 s moving window with a 1 ms time step. The results for different moving windows from different neurons were pooled when calculating the distribution. There were two far-separated groups corresponding to low-activity and persistent states, which had firing rates less than 5 $s^{-1}$ and much larger than 5 $s^{-1}$, respectively. Therefore, we chose 5 $s^{-1}$ as the firing rate threshold, but as the separation was wide, there was a broad range of options for choosing the threshold at which the separation result shows little change.

##### Persistence score

We computed a persistence score for each persistent state, assuming that a persistent state activated by the cue input to one memory engram ideally persists until it is stopped by the cue input to another memory engram, as described in the following formulae:

$$persistent\;score={\left\langle ReLU\left(x_{i}\right)\right\rangle }_{i}$$

$$x_{i}=1-\frac{\left|T_{i}^{persist}-T^{ideal}\right|}{T^{ideal}}\text{,}$$

Where ${\left\langle .\right\rangle }_{i}$ is the mean of neurons in the memory engram, *i*; ReLU is the rectified linear function, expressed as ReLU(*x*) = max(0, *x*); $T_{i}^{persist}$ is the total duration of the persistent state of the memory engram, *i*, excluding the cue input period; and $T^{ideal}$ is the time difference between the termination of the cue input for one memory and the onset of the cue input for another memory. If the persistent state lasted for $T^{ideal}$, the persistence score was 1. If the persistent state was different from $T^{ideal}$, either shorter or longer, the persistence score was less than 1. A smaller score indicated that $T_{i}^{persist}$ was further away from $T^{ideal}$.

##### Overlapping proportion

To quantify the degree of co-activation of multiple memories, we computed the overlap among the persistent states of all memory engrams using an overlapping proportion, defined as

$$Overlapping\;proportion=\frac{{⋂}_{i}T_{i}^{high}}{\underset{i}{⋃}T_{i}^{high}}\text{,}$$

where $T_{i}^{high}$ is the total time of the persistent state for the memory engram, *i*, excluding the cue input period. The overlapping proportion was bounded by 1, and a larger value indicated more co-activation of multiple memories.

##### Oscillation power

To quantify the oscillation in the simulation, we calculated the power spectrum using the fast Fourier transform of the mean-detrended average membrane potential of all excitatory neurons in the 10 s time interval after the cue input termination and normalized it to the mean of the power spectrum.

### Quantification and statistical analysis

Statistical analyses and figure generation were performed in MATALAB (MathWorks). All data were presented as the mean ± SEM, except contour, which was presented as mean. The exact number of simulation trials of each experiment was indicated in the figure legends. For the comparison of parameters including only 2 conditions, unpaired Student’s t-test was used. For the comparison the effect of 2 or 3 variables within the same graph, One-way ANOVA and Two-way ANOVA were performed respectively.

Statistical outcomes were based on p < 0.05 and displayed throughout figures as: ‘ns’ not significant, ∗p < 0.05, ∗∗p < 0.01, ∗∗∗∗p < 0.0001.

## Data Availability

• All data reported in this paper will be shared by the lead contact upon request. • All original code has been deposited at Zenodo and is publicly available as of the date of publication. The DOI is listed in the key resources table. • Any additional information required to reanalyze the data reported in this paper is available from the lead contact upon request.
