# Supplementary material for: Memory rescue and learning in synaptic impaired neuronal circuits
Source: iScience. 2023 May 29;26(7):106931. doi: 10.1016/j.isci.2023.106931 (PMC10391582; doi:10.1016/j.isci.2023.106931)
Supplement: Document S1. Figures S1–S3 and Table S1 [file mmc1.pdf]

iScience, Volume *26*

Supplemental information

Memory rescue and learning in synaptic impaired neuronal circuits

Kwan Tung Li, Daoyun Ji, and Changsong Zhou

Supplemental Figures

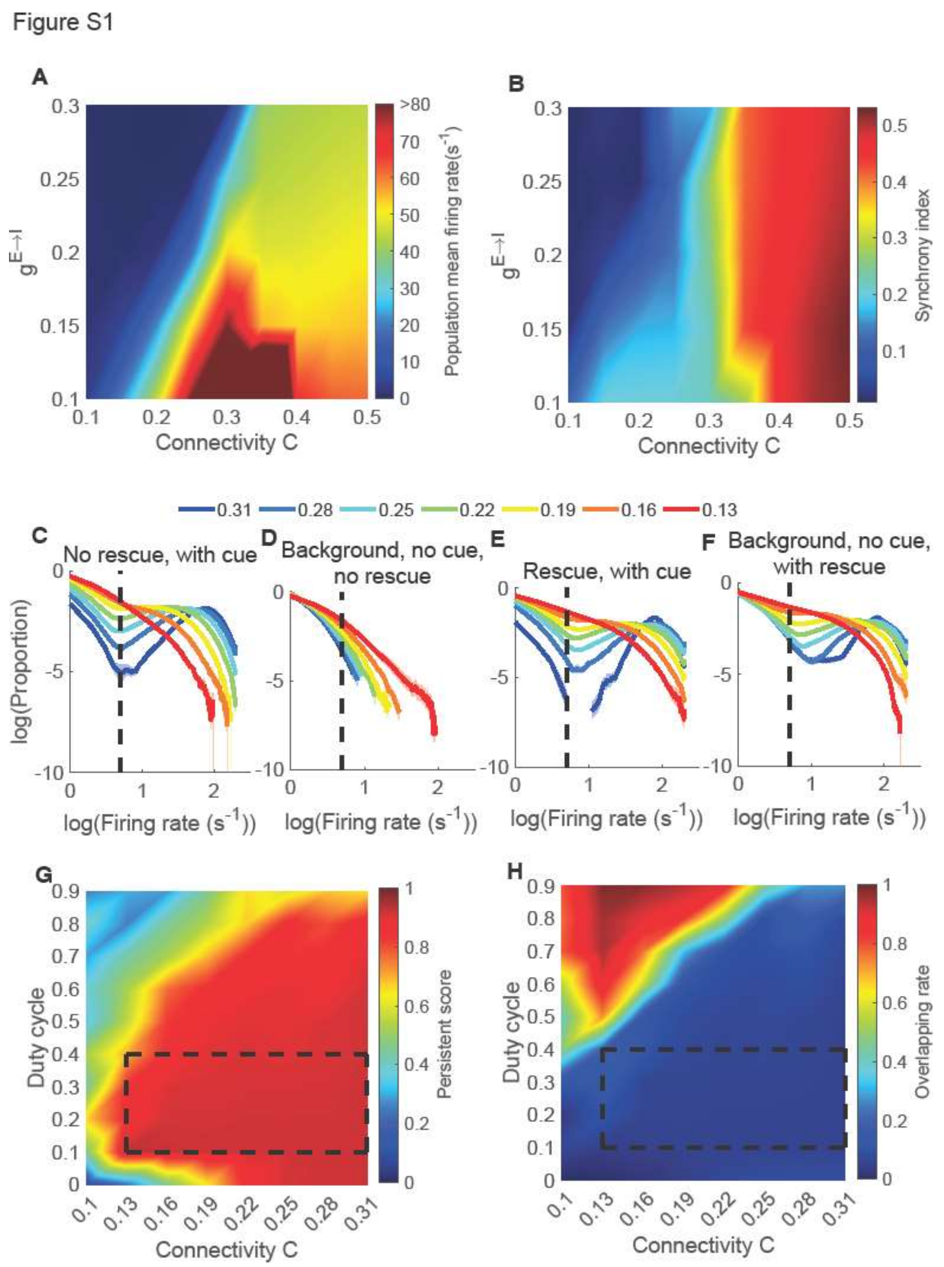

Figure S1 (related to Figure 1). Cue-induced firing properties of memory neurons change with connectivity C in a wide range of values for the synaptic weight between excitatory and inhibitory neurons $g^{E \to I}$, in the circuit without rescue stimulations.

(A, B): Population mean firing rate and synchrony index calculated in a time window from the cue termination to 10s afterwards. The increase in population mean firing rate when C reduces happens when $g^{E \to I}$ ranges from 0.1 to 0.2

and when C < 0.2, firing rates and firing synchrony are low across all $g^{E\to I}$, suggesting a robust loss of persistent states.

(C-F): Distribution of the firing rate of excitatory neurons in the network under different conditions. Sequential recall of memory engrams (within 10s after the termination of the cue input) (C). Background activity in the absence of cue input (D). Same as (C), but with rescue stimulations (40Hz 50% duty cycle) (E). Same as (D), but with rescue stimulations (40Hz 50% duty cycle) (F). The zero proportion in (E) at $C = 0.31$ causes undefined value and thus the curve is separated into two. Vertical dash line indicates firing rate at $5s^{-1}$. n=10 trials/setting. Note the log scale on x and y-axis. In (C, E), the distribution of firing rate of excitatory neurons displays a bi-modal distribution, suggesting a distinction between a background state with low rates and a persistent state with high rates, separated at ~5 s^{-1}. In (F), one memory engram may be activated spontaneously when C is high in the absence of cue and rescue stimulations.

(G, H) Persistent state (G) and Overlapping proportion at different connectivity C with different duty cycles of slow-gamma stimulations (at 40Hz). Dash rectangles indicate the duty cycle range with high persistence score and low overlapping proportion when $C > 0.13$. The result suggests that memory rescue by slow-gamma oscillations may be improved when the duty cycle of optogenetic stimulation is reduced (50% is used in the main text). Data in (A, B, G, H) are represented as mean and data in (C-F) are represented as mean ± SEM. n=10 trials/setting.

Figure S2

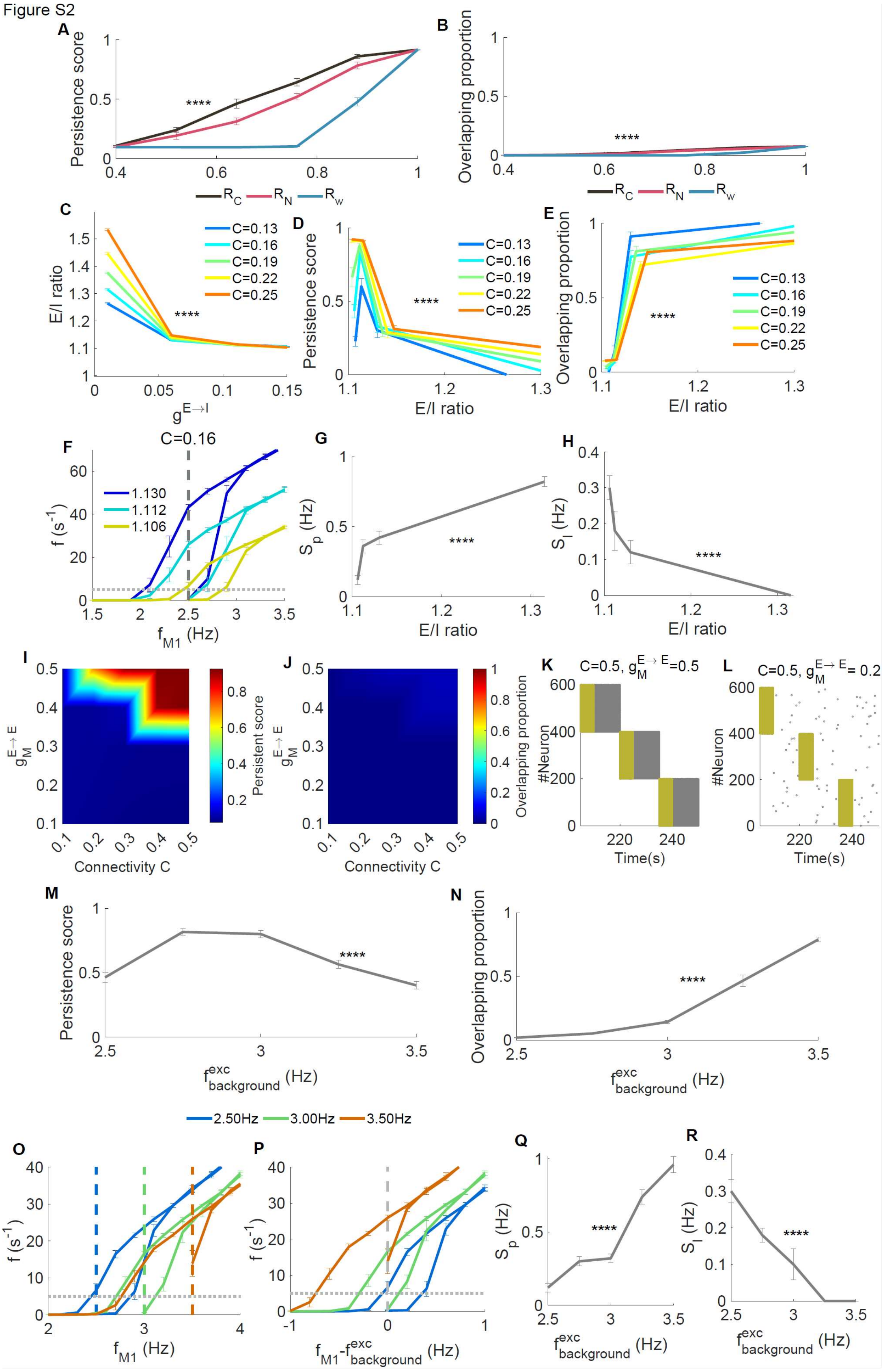
A
Persistence score
R_C R_N R_W
****
B
Overlapping proportion
R_C R_N R_W
****
C
E/I ratio
$g^{E\rightarrow I}$
C=0.13
C=0.16
C=0.19
C=0.22
C=0.25
****
D
Persistence score
E/I ratio
C=0.13
C=0.16
C=0.19
C=0.22
C=0.25
****
E
Overlapping proportion
E/I ratio
C=0.13
C=0.16
C=0.19
C=0.22
C=0.25
****
F
C=0.16
$f\ (s^{-1})$
f_{M1} (Hz)
1.130
1.112
1.106
G
S_p (Hz)
E/I ratio
****
H
S_I (Hz)
E/I ratio
****
I
$g_M^{E\rightarrow E}$
Connectivity C
Persistent score
J
$g_M^{E\rightarrow E}$
Connectivity C
Overlapping proportion
K
C=0.5, $g_M^{E\rightarrow E}$=0.5
#Neuron
Time(s)
L
C=0.5, $g_M^{E\rightarrow E}$= 0.2
#Neuron
Time(s)
M
Persistence socre
$f^{exc}_{background}$ (Hz)
****
N
Overlapping proportion
$f^{exc}_{background}$ (Hz)
****
2.50Hz
3.00Hz
3.50Hz
O
$f\ (s^{-1})$
f_{M1} (Hz)
P
$f\ (s^{-1})$
$f_{M1}-f^{exc}_{background}$ (Hz)
Q
S_p (Hz)
$f^{exc}_{background}$ (Hz)
****
R
S_I (Hz)
$f^{exc}_{background}$ (Hz)
****

Figure S2 (related to Figure 1, 2, 3). Other factors affecting memory performance.

(A-B): Effects of fraction of connections remained (R_C), neuron remained (R_N) and connection strength remained (R_w) on memory performance – Persistence score (A), Overlapping proportion (B). Each time only one of R_C, R_N and R_w varies, and the others are fixed at 1. The results suggested that neuron loss and connection strength reduction had similar effect, but with different degree of damage to memory.

(C): E/I ratio with respect to different g^{IE} at different C.

(D-E): Memory performance at different E/I ratio and C. (D): Persistence score; (E): Overlapping proportion.

(F): Firing rate of memory engram vs. E/I ratio at $C = 0.16$.

(G-H): Stability of persistent state, S_p (G), and stability of low activity state, S_l (H) with vs. E/I ratio at $C = 0.16$. the results suggested that E/I imbalance can be delineated as it has strong co-activation of memory engrams.

(I): High C and $g_M^{E \to E}$ induces high persistence score.

(J): The overlapping proportion is low among settings of different C and $g_M^{E \to E}$.

(K, L): Raster plot of $C = 0.5$ in the circuit in Figure 1A (without rescue) at (K): $g_M^{E \to E} = 0.5$; (L): $g_M^{E \to E} = 0.2$.

(M-N): Memory performance at different background input to excitatory neurons, $f_{background}^{exc}$, at C=0.16, while the background input to inhibitory neurons was maintained at $f_{background} = 2.5Hz$. (M): Persistence score; (N): Overlapping proportion.

General increase of excitability improves the persistence score when $f_{background}^{exc}$ was slightly increased, but the overlapping proportion still becomes worse.

(O): Firing rate of memory engram vs. cue inputs, f_{M1} for $M1$ at different $f_{background}^{exc}$ for non-cued neurons at C=0.16. Colored vertical dashed lines

indicated the corresponding $f_{background}^{exc}$. Increasing $f_{background}^{exc}$ moved both the bifurcation curves and $f_{M1} = f_{background}^{exc}$ to the right.

(P): Same as (O), but the colored vertical dashed lines were aligned. $f_{M1} = f_{background}^{exc}$ was moved faster than the curve in (O).

(Q-R): Stability of persistent state, S_p (Q), and stability of low activity state, S_l (R) vs. $f_{background}^{exc}$ at C=0.16. When $f_{background}^{exc}$ increased, S_p was increased, but S_l was reduced. Further increased $f_{background}^{exc}$ (>3Hz) would cause S_l to vanish to zero. The cued engram was not easy to terminate and hence had reduced the persistence score in (M); but the non-cued engrams were easily activated due to overexcitation and hence had high overlapping proportion in (N). The results suggested that increasing excitability might be another way to partially rescue memory. Data are represented mean ± SEM. n=10 trials/setting. ****: p<0.0001. In (A-F, Q, R), 2ANOVA test; In (G, H), 1ANOVA test.

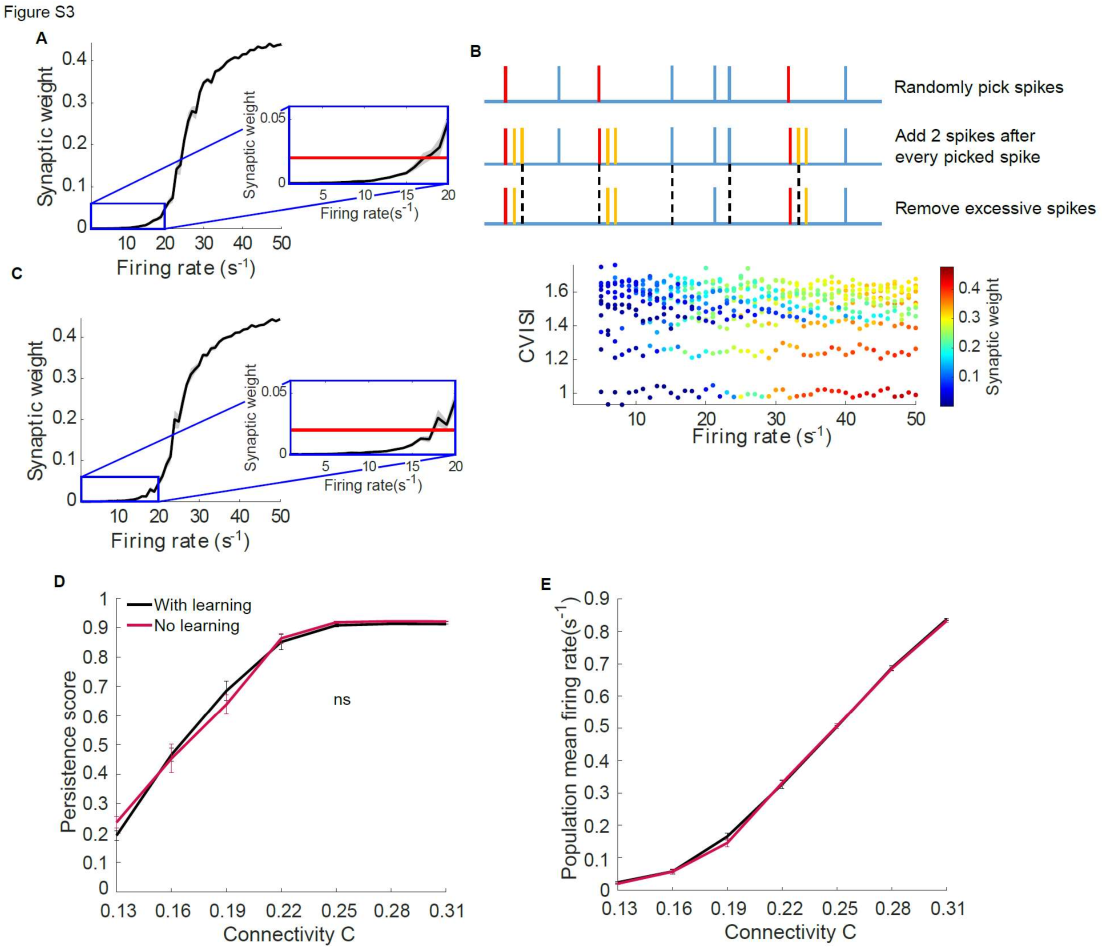

Figure S3(related to Figure 3). Factors affect synaptic weight change in learning engram and preset engram deterioration.

(A-C): Dependence of final synaptic weight after learning on firing rate and CVISIs in a 2-neuron simple model. (A): Dependence of the synaptic weight (black line) on the firing rate (the same for presynaptic and postsynaptic neurons), with CVISI=1 (Poisson spike train). Insert plot is a magnification plot of postsynaptic neuronal firing rate arranging from 0 to $10s^{-1}$. Red line in the insert indicates the synaptic weight before learning. It shows when post-synaptic firing rate, is larger (smaller) than around $10s^{-1}$, the synapse is potentiated (depressed).

(B): Dependence of the synaptic weight (black line) on the postsynaptic neuronal firing rate, with the presynaptic neuronal firing rate fixed at $50s^{-1}$ and

CVISI=1. Insert plot is a magnification plot of postsynaptic neuronal firing rate arranging from 0 to $10s^{-1}$. Red line indicates the synaptic weight before learning. n=10 trials/setting.

(C): (Top): Illustration of spike train generation to manipulate CVSI. 1) Pick spikes randomly (red lines) with probability P_{pikc} from Poisson spike train. 2) Add 2 spikes (orange lines) after every picked spike. 3) Remove spikes randomly (black dash lines) to maintain the number of spikes as the original Poisson spike train. (Bottom): Synaptic weight (color coded) depending on the firing rate (the same for presynaptic and postsynaptic neurons) and CVSI. Change of CVISI is obtained by spike train manipulation in (B). The difference in synaptic weights is because large CVISI implies there is temporally high firing rate, which would activate the heterosynaptic depression to restrict potentiation by triplet synaptic plasticity. The dependence on CVISI is more important than the dependence on firing rate in the study of learning because the firing rate is at high value (>30 Hz) during learning.

(D-E): Preset engram deterioration. Persistence score (D) and Population mean firing rate (E) of preset engrams during recall with (Black) and without (red) learning before recall. Although synaptic weights in the preset engrams are modestly impacted by learning (Figure 3C), the effect is not strong enough to further impair the recall of preset engrams. Data are represented as mean ± SEM. n=10 trials/setting. ns, $p > 0.05$ in 2ANOVA in (D, E).

Table S1 Parameters used in the neural circuit model. Related to STAR Methods.

Description	Symbol	Value
Numbers of E, I neurons	n_E, n_I	2000,400
Default firing rate of background input and stimulus input (per connection)	$f_{background}, f_{stimuls}$	2.5Hz, 12.5Hz
Axonal delay	τ_l	1ms
leakage potential	V_L	-70mV
Threshold potential	V_{th}	-50mV
Rest potential	V_{rest}	-55mV
Membrane time constant for E, I neurons	τ^E, τ^I	20ms,10ms
Refractory period for E, I neurons	$t^E_{refratory}, t^I_{refratory}$	2ms,1ms
Reversal potential of E, I neurons	E^E, E^I	0mV，-70mV
Leakage conductance	g_L	10nS
Input conductance from background to E, I neurons	$g^{O\to E}, g^{O\to I}$	0.05, 0.08 (normalized by g_L)
Input conductance from E to E, I to E, E to I, I to I and preset within engram	$g^{E\to E}, g^{I\to E}, g^{E\to I}, g^{I\to I}$, $g_M^{E\to E}$	0.02, 0.9, 0.15, 0.48, 0.5 (normalized by g_L)
Decay time constant of AMPA, GABA, and NMDA current	$\tau_d^{AMPA}, \tau_d^{GABA}, \tau_d^{NMDA}$	3ms, 8ms, 100ms
Amplitude of synaptic time course	$\mu^{AMPA}, \mu^{NMDA}, \mu^{GABA}$	1.1, 0.8, 1
Rising time constant of AMPA, GABA, NMDA current	$\tau_r^{AMPA}, \tau_r^{GABA}, \tau_r^{NMDA}$	0.5ms, 0.5ms, 0.5ms
Facilitation time constant in STP	τ_F	1500ms
Depression time constant in STP	τ_D	200ms

Initial neural transmitter release probability in STP	U	0.2
Learning rate for long-term potentiation	A	0.006
Learning rate for long-term depression	B	0.006
Learning rate for heterosynaptic plasticity	β	1×10^{-6}
Preferred synaptic weight	$\widetilde{w}$	0.02
Transmitter plasticity strength	δ_1	$1\text{x}10^{-6}$
Characteristic time constant for synaptic trace	τ_{STDP}	20ms
Characteristic time constant for slow synaptic trace	τ_{STDP_slow}	100ms
